# The role of entry screening in case finding of tuberculosis among asylum seekers in Norway

**DOI:** 10.1186/1471-2458-10-670

**Published:** 2010-11-04

**Authors:** Ingunn Harstad, Geir W Jacobsen, Einar Heldal, Brita A Winje, Saeed Vahedi, Anne-Sofie Helvik, Sigurd L Steinshamn, Helge Garåsen

**Affiliations:** 1 Department of Public Health and General Practice, Norwegian University of Science and Technology, MTFS, NO-7491 Trondheim, Norway; 2 Norwegian Institute of Public Health, PO box 4404 Nydalen, NO-0403 Oslo, Norway; 3 Department of Pulmonary Medicine, Ullevål University Hospital, NO-0407 Oslo, Norway; 4 Department of Circulation and Medical Imaging, Norwegian University of Science and Technology, NO-7491 Trondheim, Norway; 5 Department of Pulmonary Medicine, St. Olavs Hospital, Trondheim University Hospital, NO-7006 Trondheim, Norway; 6 City of Trondheim, Department of Health and Social Welfare, NO-7004 Trondheim, Norway

## Abstract

**Background:**

Most new cases of active tuberculosis in Norway are presently caused by imported strains and not transmission within the country. Screening for tuberculosis with a Mantoux test of everybody and a chest X-ray of those above 15 years of age is compulsory on arrival for asylum seekers.

We aimed to assess the effectiveness of entry screening of a cohort of asylum seekers. Cases detected by screening were compared with cases detected later. Further we have characterized cases with active tuberculosis.

**Methods:**

All asylum seekers who arrived at the National Reception Centre between January 2005 - June 2006 with an abnormal chest X-ray or a Mantoux test ≥ 6 mm were included in the study and followed through the health care system. They were matched with the National Tuberculosis Register by the end of May 2008.

Cases reported within two months after arrival were defined as being detected by screening.

**Results:**

Of 4643 eligible asylum seekers, 2237 were included in the study. Altogether 2077 persons had a Mantoux ≥ 6 mm and 314 had an abnormal chest X-ray. Of 28 cases with tuberculosis, 15 were detected by screening, and 13 at 4-27 months after arrival. Abnormal X-rays on arrival were more prevalent among those detected by screening. Female gender and Somalian origin increased the risk for active TB.

**Conclusion:**

In spite of an imperfect follow-up of screening results, a reasonable number of TB cases was identified by the programme, with a predominance of pulmonary TB.

## Background

In recent years most new tuberculosis (TB) cases in Norway have occurred among immigrants from high incidence countries. Rarely, new cases are due to transmission within the country [1].

Low incidence countries have diverse policies on entry screening of immigrants from high incidence countries. These range from no screening at all, to pre-immigration screening or screening after arrival [2-4]. There is an ongoing discussion about the content and effectiveness of different screening programmes to control tuberculosis [5,6]. Studies of screening of tuberculosis among immigrants have given TB prevalences that range from 0.1-1.2% [7-10], that can be due to differences in the characteristics of the populations and the screening programmes.

Previous studies have shown differences between cases detected by or outside the screening programme. Cases found by screening had fewer symptoms and fewer positive cultures [11,12]. Other studies have shown that a high number of TB cases have been detected in immigrant populations several years after immigration [5,6,13].

Risk factors for tuberculosis can be divided into the risk for being infected and the risk for developing disease. Typical risk factors for being infected are for instance poverty and a history of being incarcerated. Country of origin could be related both to risk for being infected and for developing disease. Recent infection, reduced immunity from other diseases like HIV or medical treatment, intake of alcohol, smoking, and body weight loss are related to the risk for developing disease [14-19].

### The tuberculosis screening programme in Norway

Screening of all asylum seekers for tuberculosis is mandatory on arrival in Norway. The aims of the screening are to diagnose and treat active TB, and to diagnose cases with latent TB infection, either for treatment or observation for development of active TB in a three year follow-up programme [20]. However, the programme does not clearly differentiate between screening for active and latent disease.

The National Reception Centre outside Oslo performs a Mantoux test of everybody and a chest X-ray of persons above the age of 15 years. According to the guidelines, person who report symptoms suggestive of TB, or have an abnormal chest X-ray, shall be referred to the Central TB Clinic in Oslo for assessment of TB disease. Persons with a Mantoux test ≥ 15 mm shall be referred to the specialist health care for evaluation of TB disease or infection by a chest physician or an internist. Everyone with a Mantoux test result of 6-14 mm shall be followed up by primary health care (PHC). Whenever definite risk factors are identified, the patient shall be referred to a specialist [21]. However, diagnosis of latent TB remains a challenge and recommendations for diagnosis and follow-up are ambiguous. TB disease is diagnosed on the basis of symptoms, clinical examinations, chest X-ray and positive bacteriology. In Norway, only specialists are allowed to start TB treatment. Whenever a TB diagnosis is confirmed and/or treatment for TB is started, a nominal notification is sent to the National Tuberculosis Register. After screening in the National Reception Centre, asylum seekers move to other centres for asylum seekers throughout the country or to private accommodations.

Surveillance data for TB in Norway, can not distinguish asylum seekers from immigrants. An earlier Norwegian study on chest X-ray results from entry screening of asylum seekers, concluded that an improved follow-up of persons with an abnormal X-ray is necessary [22]. Thus, they found that 11(14%) of the cases that were not diagnosed by the arrival screening but diagnosed later, had been inadequately followed up. A previously published paper of the present cohort showed that 194/314 (62%) subjects with an abnormal X-ray had been seen by an internist, while altogether 758/2237 (34%) with a positive Mantoux test and/or an abnormal X-ray had been followed up at the primary health care level. Inadequate handling of screening results was found at all levels of care [23].

The aims of the present study are to assess the effectiveness of the tuberculosis entry screening programme for diagnosing active TB in a cohort of asylum seekers and to characterize all cases of active tuberculosis. Further we aim to compare patients who were diagnosed by the screening programme with those who were detected later.

## Methods

### Study population

All asylum seekers aged 18 years or over, and arriving at the National Reception Centre from January 2005 - June 2006 were screened with a chest X-ray and a Mantoux test. They were included in the study if they were available for follow-up and had an abnormal chest X-ray, a Mantoux test ≥ 6 mm and/or a positive QuantiFERON^® ^TB Gold (QFT) test. Due to the study design it was not possible to include children below 18 years of age in the study. In case they needed specialist medical attention or care, they were referred to paediatricians and not to chest physicians.

Of 5112 asylum seekers screened during the study period, 469 could not be followed up because they were either deported, deceased or had no registered address, leaving a total cohort of 4643 persons. Of the remaining 4643 (called the total cohort) 2293 had positive study inclusion criteria. However, 56 were lost from the National Reception Centre and hence, 2237 study participants were included for follow-up [23].

An abnormal chest X-ray was defined as parenchymal or pleural pathology, or calcifications. The X-rays were interpreted by two independent readers and later recoded for this study. A positive Mantoux test was defined as ≥ 6 mm (PPD: RT 23, 2TU from SSI, Copenhagen, Denmark). An interferon-γ release assay test: (QuantiFERON^® ^TB Gold in-tube test, Cellestis Ltd, Carnegie, Victoria, Australia, QFT) was done as a separate study on a subgroup of 912 participants [24].

### Collection of study information

Subject demographics and screening results were collected from the data files at the National Reception Centre. Further information was collected by one registration form that was sent to the public health care official in the municipality to where the asylum seeker had moved. If any person in question had moved on, the same form was sent to the appropriate municipality. If the PHC form indicated that a referral had taken place, a specialist form was sent to the appropriate internist. A specialist form was also sent to the Central TB Clinic when we received information from the National Reception Centre that an asylum seeker had either been referred there or had an abnormal X-ray. Both forms collected recorded information about assessment by health care personnel, plans for follow-up and the reason for referral of the individual. In addition we collected information about current addresses or relocations of study participants.

A match by name and date of birth for everyone in the study population and the National Tuberculosis Register was done in May 2008. The register holds information about localisation of disease, culture results, the individual physician who had reported the case, and some background information about the specialist referral.

Cases reported to the National Tuberculosis Register within two months after arrival were categorized as detected by screening in contrast to those who were detected later.

### Data registration and analyses

Study forms were scanned and entered into the SPSS program (SPSS for Windows, version 16, Chicago, Ill, USA). Comments and administrative information were coded manually and entered to the same data file.

The study group and the TB cases were described by proportions and 95% confidence intervals (CI). Time from arrival to notification to the Tuberculosis Register was approximated to the closest number of total months.

The primary outcome diagnosed as active tuberculosis or not, was analysed with logistic regression. The assessed regressors were stratified age, gender, country of origin (Somalian or not), marital status (married or not), Mantoux (≥ 10 or not, and ≥ 15 or not) and X-ray (abnormal or not). Variables in the univariate analysis that changed the odds ratio (OR) of the other variables with 0.2 or more were entered into the multivariable analysis. The model was checked for correlations and interactions.

Due to low number of cases, the secondary outcome; i.e cases that were detected by screening or not, was studied only by univariate logistic regression. Additional regressors for the secondary outcome were a diagnosis of pulmonary TB (as opposed to extra-pulmonary TB) (yes/no) and a positive culture (yes/no).

### Ethics

The Regional Committee for Medical Research Ethics approved the study. The Norwegian Data Inspectorate, Directorate for Health and Social Affairs, Ministry of Labour and Social Inclusion, and the Research Committee at Ullevaal University Hospital all gave their permission.

## Results

There were 3222 (69%) males and 3333 (72%) were between 18 to 34 years in the total cohort. The top five countries of origin for asylum seekers were Iraq, Somalia, Russia, Afghanistan and Serbia and Montenegro with 2434 (52%) individuals [23].

Among the 2237 asylum seekers who fulfilled the inclusion criteria and were included in the study group, 1563 (70%) were males and 1447 (65%) were 18-34 years of age (Table 1). An abnormal X-ray was found in 314 persons, 2077 had a Mantoux ≥ 6 mm and 27 were included because of a positive QFT only. Compared to those not included, the study group had significant fewer persons in the lowest and more in the middle and oldest age group, more were married, came from Somalia, and had a BCG scar (data not shown).

**Table 1:** Numbers, percents and CI by demographics and screening results for study group and TB cases

	Groups	Study group N = 2237		TB cases N = 28	
		**Numbers (%)**	**95% CI**	**Numbers (%)**	**95% CI**

Age groups	18-34 years	1447 (65)	63-67	22 (79)	63-94
	35-49 years	656 (29)	27-31	4 (14)	1-27
	>50 years	134 (6)	5-7	2 (7)	-2-17
Gender	Males	1563 (70)	68-72	13 (46)	28-65
	Females	674 (30)	28-32	15 (54)	35-72
Marital status	Married	1034 (46)	44-48	10 (36)	18-53
	Not married	1203 (54)	52-56	18 (64)	47-82
The five most frequent countries	Iraq	224 (10)	9-11	1 (3.6)	-3-10
	Somalia	457 (20)	19-22	15 (54)	35-72
	Russia	182 (8)	7-9	4 (14)	1-27
	Afghanistan	166 (7.4)	6-9	1 (3.6)	-3-10
	Serbia and Montenegro	149 (6.7)	6-8	2 (7.1)	-2-17
Mantoux test	<6 mm	146 (6.5)	6-8	1 (3.6)	-3-10
	6-9 mm	680 (30)	28-32	2 (7.1)	-2-17
	10-14 mm	760 (34)	32-36	8 (29)	12-45
	≥ 15 mm	637 (28)	27-30	12 (43)	25-61
Abnormal X-ray		314 (14)	13-15	16 (57)	39-75

### Cases of tuberculosis

By end May 2008, 28 cases of tuberculosis were diagnosed. No case had recieved treatment for latent tuberculosis after arrival in Norway. The median time from arrival until the National Tuberculosis Register was notified was two months (range 0-27). The diagnosis was made within two and six months of arrival in 15 and 17 individuals, respectively. Eight patients were diagnosed later than one year after arrival (Table 2).

**Table 2:** TB cases diagnosed by screening or not by demographics, screening results and TB Register report

Case number	Diagnosed by screening	Arrival to diagn. (months)	Age on arrival (years)	Gender	Married	Country of origin	Mantoux result on arrival (mm)	X-ray findings on arrival	Culture results on diagnosis	Localisation of disease
1	Yes	0	25	F	Yes	Somalia	9	Parenchymal	Positive	Pulmonary
2	Yes	0	28	F	Yes	Somalia	No	Parenchymal	Positive	Pulmonary
3	Yes	0	24	M	No	Afghanistan	12	Parenchymal	Positive	Pulmonary
4^a^	Yes	0	24	F	No	Somalia	2	Normal	No	Pulmonary
5	Yes	0	43	M	Yes	Russia	18	Parenchymal	Positive	Pulmonary
6	Yes	0	30	F	No	Somalia	16	Parenchymal	Positive	Lymphatic
7	Yes	0	25	M	No	Somalia	No	Parenchymal	Positive	Pulmonary
8	Yes	0	31	M	No	Serbia/Montenegro	No	Parenchymal	Positive	Pulmonary
9	Yes	1	49	F	No	Somalia	17	Parenchymal	Positive	Pulmonary
10	Yes	1	23	M	No	China	15	Parenchymal	Positive	Pulmonary
11	Yes	1	22	M	No	Serbia/Montenegro	No	Pleura	Positive	Pulmonary
12	Yes	1	24	F	No	Somalia	10	Normal	Positive	Unknown
13	Yes	2	50	M	Yes	Iraq	No	Parenchymal	Negative	Pulmonary
14	Yes	2	33	M	No	Algeria	10	Parenchymal	Positive	Pulmonary
15	Yes	2	29	F	Yes	Congo rep	20	Normal	Positive	GI/periton.
16	No	4	19	M	No	Somalia	15	Normal	Positive	Lymphatic
17	No	5	51	F	No	Yemen	15	Pleural	No	Pulmonary
18	No	8	26	M	No	Russia	8	Parenchymal	Positive	Pulmonary
19	No	10	37	M	Yes	Russia	20	Normal	No	Pulmonary
20	No	11	21	F	Yes	Somalia	11	Normal	Positive	Lymphatic
21	No	15	26	F	No	Syria	19	Normal	Positive	Pulmonary
22	No	15	26	F	No	Somalia	13	Normal	Positive	CNS
23	No	15	23	F	Yes	Somalia	13	Normal	Positive	Pulmonary
24	No	16	45	F	No	Somalia	17	Normal	No	Unknown
25	No	17	25	F	No	Somalia	20	Parenchymal	Positive	Lymphatic
26	No	20	31	M	Yes	Russia	14	Normal	Positive	Pulmonary
27	No	20	25	M	Yes	Somalia	15	Parenchymal	Positive	GI/periton.
28	No	27	24	F	No	Somalia	14	Normal	Positive	Lymphatic

^a^This case was included because of a positive QFT test

Twenty two cases had *M. tuberculosis *and one case had *M. Africanum *confirmed by culture. Of the remainder, one was culture negative and four had unknown bacteriology. There were 18 individuals with pulmonary and eight with extra-pulmonary TB, while the localization was unknown in two individuals. Somalian origin was associated with extra-pulmonary TB (data not shown). Of 15 women with tuberculosis, 12 were from Somalia. The Somalian females median age was 25 years (range 21-49). Mantoux ≥ 15 mm on arrival was found in 12 (43%) cases and Mantoux ≥ 10 mm in 20 (69%) cases (Table 2). Lack of a BCG vaccination scar was not associated with active TB (data not shown).

In a univariate analysis for all cases of active TB, a positive association was found with a positive chest X-ray, Mantoux ≥ 15 mm, Mantoux ≥ 10 mm, female gender, and Somalian origin (Table 3). In adjusted logistic regression models, gender and country of origin were entered into the model. A positive association with active TB was found for female gender and Somalian origin when either Mantoux ≥ 10 mm, or Mantoux ≥ 15 mm, or a positive chest X-ray were included (Table 4). However, in the adjusted model neither Mantoux ≥ 10 mm nor Mantoux ≥ 15 mm was associated with active TB.

**Table 3:** Univariate analysis of associations between demographics, screening results and active tuberculosis

			Univariate analysis	
	**Cases** **N = 28**	**Non-cases** **N = 2209**	**OR**	**95%CI**

Age 18-34	22	1425	1(ref)	
Age 35-49	4	652	0.42	0.14-1.2
Age ≥ 50	2	132	1.6	0.5-5.3
Male	13	1550	1(ref)	
Female	15	659	2.7	1.3-5.7
Unmarried	18	1016	1(ref)	
Married	10	1193	0.64	0.30-1.4
Not from Somalia	13	1767	1(ref)	
Somalia	15	442	4.6	2.2-9.8
Mantoux 6-9 mm	2	678	0.17	0.04-0.73
Mantoux ≥ 10 mm	20	1377	4.0	1.2-13.4
Mantoux ≥ 15 mm	12	625	2.8	1.2-6.3
Abnormal X-ray	16	298	8,6	4.0-18.3

**Table 4:** Multivariable analysis of associations between demographics, screening results and active tuberculosis

	Mantoux ≥ 10 mm		Mantoux ≥ 15 mm		Abnormal X-rays	
**Variables**	**OR**	**95% CI**	**OR**	**95% CI**	**OR**	**95% CI**

Male	1(ref)		1(ref)		1(ref)	
Female	2.9	1.2-6.8	2.8	1.2-6.6	2.6	1.2-5.7
Not from Somalia	1(ref)		1(ref)		1(ref)	
Somalia	4.0	1.7-9.4	4.2	1.8-9.8	4.7	2.2-10.1
Mantoux ≥ 10 mm	2.9	0.8-9.9	**-----**	**-----**	**-----**	**-----**
Mantoux ≥ 15 mm	**-----**	**-----**	1.9	0.8-4.4	**-----**	**-----**
Abnormal X-ray	**-----**	**-----**	**-----**	**-----**	9.7	4.5-21.2

Three multivariable models where included variables are Somalia, gender, and either Mantoux ≥ 10 mm, Mantoux ≥ 15 mm or an abnormal X-ray

### The yield of screening

Fifteen cases were detected within two months after arrival. Twelve of the cases had positive X-ray findings when they arrived and 13 had symptoms of TB at the time of diagnosis. There were twelve cases of pulmonary and two with extra-pulmonary TB and one with unknown localization. Thirteen had a positive culture (Table 2 and 5).

**Table 5:** Univariate analysis: cases detected by screening or not, by demographics, screening results, and characteristics

	Detected by screening n = 15	Missing values	Not detected by screening n = 13	Missing values	OR	95% CI
18-34 years	12		10		1.2	0.20-7.3
≥ 35 years	3		3			
						
Females	7		8		0.55	0.12-2.5
						
Somalia	7		8		0.55	0.12-2.5
						
Mantoux >10	8 (2)	5	12 (1)	0	0.33	0.03-4.3
Mantoux >15	5 (5)	5	7 (6)	0	0.86	0.16-4.5
						
Abnormal X-rays	12		4		9.0	1.6-50.7
TB pulm	12 (2)	1	6 (6)	1	6.0	0.92-39.2
Culture positive	13 (1)	2	10 (0)	3	0.001	

Brackets indicates negative numbers when there are some missing values

All cases diagnosed by screening were reported from hospitals in the Oslo area. Of the 12 cases with an abnormal X-ray on arrival, six had visited the Central TB Clinic where all should have been referred according to the guidelines.

Of the 13 cases that were not detected through the screening programme, two cases were diagnosed from three to six months after arrival, (four and five months respectively), one with pulmonary and one with extra-pulmonary TB. One of the cases was confirmed by culture. None of them had been examined at the Central TB Clinic. Still, one had reported symptoms on arrival.

The remaining eleven cases were diagnosed from eight to 27 months after arrival. Five of them had pulmonary and five other extra-pulmonary TB. In the last case the localisation was unknown. Nine of these 11 patients had the diagnosis confirmed by culture. Moreover, three patients had an abnormal X-ray on the arrival screening and 12 patients had a Mantoux ≥ 10 mm (Table 2).

All three cases who had an abnormal X-ray on arrival and were detected later than six months after arrival, had been examined at the Central TB Clinic just after arrival, but were judged not to have TB disease at that time. They were advised to have additional follow-up if there was a future doubt about the diagnosis. Thus, they most likely developed the disease later. Ten of the 11 late cases were diagnosed at different hospitals around the country. They had all been referred from PHC due to clinical symptoms. One case diagnosed after ten months had been referred from PHC to specialist for assessment of screening results following a move to an asylum seekers centre in the community.

In univariate logistic regression of cases detected by screening or not, for an abnormal X-ray the OR was 9.0 (1.6-50.7). The distinction between pulmonary TB and extra-pulmonary TB detected by screening gave an OR of 6.0 (0.9-39.2). However, the latter OR was not significant (table 5). We found no other associations between time of diagnosis and either demographics or screening results.

## Discussion

The yield of the Norwegian TB screening programme that was aimed at a high risk population and defined as cases detected within 2 months of arrival was 15/2237 (0.7%) cases. Our results are comparable to that of other similar studies where the prevalences ranged from 0.1.-1.2% [7-10].

Two cases were diagnosed between three and six months after arrival and were probably missed by the screening. The other 11 cases were not diagnosed as a part of the screening programme. Cases identified by screening more often had abnormal X-rays on arrival. Female gender and Somalian origin increased the risk for TB disease.

A major objective of the screening programme is to diagnose pulmonary TB as early as possible to minimize risk of transmission to others within the country. This seemed to have been the case since 12 of the 15 cases detected had pulmonary TB. Through the match with the Tuberculosis Register we detected several cases diagnosed at different hospitals in the Oslo area. However, we could not trace any information about referrals for these patients through the files at the National Reception Centre or the study forms. Previously we have reported that 62% of persons with an abnormal X-ray on screening and 70% of those with parenchymal findings had been seen by a specialist [23]. Thus, we have reasons to believe that more cases had been referred to specialist than we were able to identify through our study.

A pertinent question is whether cases that were detected later than two months after arrival could have been detected earlier or prevented. One individual who was diagnosed four months after arrival had been ill since arrival. A second individual diagnosed five months after arrival, was found through contact tracing and had symptoms at the time of referral. However, we do not know the time from symptoms to diagnosis for the remaining individuals. All 11 patients who were detected later than six months after arrival had either abnormal X-rays or a positive Mantoux test at entry. According to the guidelines they should have been referred to a specialist or examined in PHC. Seven of them should have been, but were not referred to specialist to reach a firm conclusion about entry findings. We believe that some of these cases could have been detected with active TB earlier or been given treatment for latent tuberculosis. On the other hand, the Norwegian guidelines give no definite recommendations for treatment for latent TB. Previously we have published that as few as 30/2237 (1.3%) individuals of the same cohort were treated for latent tuberculosis [25].

Asylum seekers were not interviewed about symptoms on arrival, and registration of symptoms seems to be incidental. Possibly, a medical interview could have raised suspicion in some of the cases, particularly the ones with extra-pulmonary TB. On the other hand, screening for TB in a Brazilian prison showed that 1/3 of the TB cases denied any symptoms during an initial interview, and several patients with a cough of more than three weeks duration considered that they had a "normal" cough [26].

As more cases found through screening in our study had abnormal X-rays and tended to have more pulmonary TB, an abnormal X-ray was the main indication for an immediate follow-up. There were no differences in the proportion with positive cultures between the groups diagnosed inside or outside the screening programme. This could have been a spurious finding and due to low numbers. Others have reported that cases detected outside the screening programme showed an increase in the number of positive cultures [11].

Other studies have also shown that regardless of any arrival screening there is an increased risk for active TB for several years after arrival. However, all these late cases have been interpreted as incident ones [5,6,27]. This is particularly the case for extra-pulmonary TB [6].

The association of an increased risk for active TB in asylum seekers of Somalian origin is in accordance with the findings of several other studies [5,22,28,29]. Similarly, the association between Somalian origin and extra-pulmonary TB, has also been shown in a previous Norwegian study [30].

In our study females had a significant increased risk for active TB. Further, 12 of the 15 females with tuberculosis were from Somalia. We have no information whether females from Somalia arriving in Norway have other risk factors. Low numbers in the present study or other health seeking behaviours in their home country may explain why our results differ from other studies from developing countries where males have higher risk [31]. However, studies from Western countries at the time when TB was more prevalent, showed an increased risk for females of fertile age [31].

We have analysed data from the arrival screening of a complete cohort of asylum seekers and matched subjects with positive screening information with data from the National Tuberculosis Register. However, we did not match those without positive screening results as they are not supposed to be followed up. Moreover, we have no data on the several study subjects who had left Norway during the study period. This may have influenced the number of people who were available for analysis and made exact estimating of incidences impossible. Children below 18 were not included in the study and our conclusions can not be applied to them.

Asylum seekers frequently change their name and/or date of birth which may have given an artificial reduction in the matching procedure with the National Tuberculosis Register. Such changes of demographic data may have resulted in underreporting and incomplete responses on our study forms.

Smear results were inavailable for analysis because of the limited numbers that were reported to the National Tuberculosis Register.

Only persons with an abnormal X-ray or where data stated that they had been referred from the National Reception Centre to a specialist, were matched with the Central TB Clinic. This procedure could have led to some underreporting of cases seen by specialist because a similar match was not done with other hospitals.

We defined patients who were diagnosed within two months after arrival as detected by the screening programme. Hence, misclassification of the cases may have led to over - as well as underestimation of the yield of the programme. In most cases, the files at the Tuberculosis Register gave no clear answer whether the clinical examination was the result of screening or not. Nor did they report the length of time between symptom onset and the TB diagnosis. Possibly, some late cases may have been reinfected after arrival to Norway, but infection rate within the country is generally quite low [1].

## Conclusions

More patients were referred to specialist and more cases found than we were able to detect from the follow-up study. Still, in spite of an imperfect follow-up of screening results, 15 of 17 TB cases (88%) were found through screening, most of whom had pulmonary TB. As a consequence of this study, we will underscore that an immediate follow-up of everyone who has an abnormal X-ray should be verified by a quality assurance system. In cases of latent TB, treatment should always be considered. Special measures for all asylum seekers from Somalia should also be considered.

Further studies are needed to assess cost-effectiveness of the screening programme.

## List of abbreviations used

TB: Tuberculosis; QFT: QuantiFERON^® ^TB Gold; PHC: Primary Health Care; CI: Confidence Interval; OR: Odds Ratio

## Competing interests

The authors declare that they have no competing interests.

## Authors' contributions

This paper is part of a PhD project "Tuberculosis infection and disease among asylum seekers in Norway". IH has done most of the work under supervision of GWJ, EH, and HG who all participated in the planning, implementation, analysis and writing process. SLS also supervised the implementation, analysis and writing process. BAW contributed with input in the implementation and writing process, information about the QFT study and matching with the National Tuberculosis Register. SV was responsible for the data collection at the Central Tuberculosis Clinic and contributed in the writing process. ASH supervised the data analysis and contributed in the writing process. All authors have read and approved the final manuscript.

## Pre-publication history

The pre-publication history for this paper can be accessed here:

http://www.biomedcentral.com/1471-2458/10/670/prepub

## References

[B1] Dahle UR Eldholm V Winje BA Mannsaker T Heldal E Impact of immigration on the molecular epidemiology of Mycobacterium tuberculosis in a low-incidence country Am J Respir Crit Care Med 2007 176 9 930 935 10.1164/rccm.200702-187OC 17673698

[B2] Coker R Bell A Pitman R Hayward A Watson JM Screening programmes for tuberculosis in new entrants across Europe Int J Tuberc Lung Dis 2004 8 8 1022 1026 15305488

[B3] Coker R Bell A Pitman R Zellweger J-P Heldal E Hayward A Skulberg A Bothamley G Whitfield R de Vries G Tuberculosis screening in migrants in selected European countries shows wide disparities Eur Respir J 2006 27 4 801 807 10.1183/09031936.06.00104305 16585088

[B4] Bothamley GH Ditiu L Migliori GB Lange C Active case finding of tuberculosis in Europe: a Tuberculosis Network European Trials Group (TBNET) survey Eur Respir J 2008 32 4 1023 1030 10.1183/09031936.00011708 18550615

[B5] Lillebaek T Andersen ÅB Dirksen A Smith E Skovgaard LT Kok-Jensen A Persistent high incidende of tuberculosis in immigrants in a low-incidence country Emerg Infect Dis 2002 8 7 679 684 12095434 10.3201/eid0807.010482 PMC2730343

[B6] Marks G Bai j Stewart G Simpson S Sullivan E Effectiveness of postmigration screening in controlling tuberculosis among refugees: a historical cohort study, 1984-1998 Am J Public Health 2001 91 11 1797 1799 10.2105/AJPH.91.11.1797 11684606 PMC1446881

[B7] Van den Brande P Uydenbrouck M Vermeire P Demedts M Tuberculosis in asylum seekers in Belgium Eur Respir J 1997 10 610 614 9072993

[B8] Mathez C Bangala Y Bady P Zellweger J-P Active screening for pulmonary tuberculosis by chest x-ray among immigrants at the Swiss border Swiss med wkly 2007 137 649 654 18027111 10.4414/smw.2007.11858

[B9] Harling R Pearce M Chandrakumar M Mueller K Hayward A Tuberculosis screening of asylum seekers: 1 years'experience at the Dover Induction Centres Public Health 2007 121 822 827 10.1016/j.puhe.2007.02.019 17645899

[B10] Callister MEJ Barringer J Thanabalasingam ST Gair R Davidson RN Pulmonary tuberculosis among political asylum seekers screened at Heathrow Airport, London, 1995-9 Thorax online 2002 57 152 156 10.1136/thorax.57.2.152 PMC1746236 11828046

[B11] Laifer G Widmer AF Simcock M Bassetti S Trampuz A Frei R Tamm M Battegay M Fluckiger U TB in a low-incidence country: differences between new immigrants, foreign-born residents and native residents Am J Med 2007 120 4 350 356 10.1016/j.amjmed.2006.10.025 17398230

[B12] Verver S Bwire R Borgdorff MW Screening for pulmonary tuberculosis among immigrants: estimated effect on severity of disease and duration of infectiousness Int J Tuberc Lung Dis 2001 5 5 419 425 11336272

[B13] Creatore MI Lam M Wobeser WL Patterns of tuberculosis risk over time among recent immigrants to Ontario, Canada Int J Tuberc Lung Dis 2005 9 6 667 672 15971395

[B14] Van Burg JL Verver S Borgdorff MW The epidemiology of tuberculosis among asylum seekers in the Netherlands: implication for screening Int J Tuberc Lung Dis 2003 7 2 139 144 12588014

[B15] Lee MS-N Leung C-C Kam K-M Wong M-Y Leung MC-M Tam C-M Leung EC-C Early and late tuberculosis risks among close contacts in Hong Kong Int J Tuberc Lung Dis 2008 12 3 281 287 18284833

[B16] Landry J Menzies D Preventive chemotherapy. Where has it got us? Where to go next? Int J Tuberc Lung Dis 2008 12 12 1352 1364 19017442

[B17] Lonroth K Williams BG Stadlin S Jaramillo E Dye C Acohol use as a risk factor for tuberculosis: a systematic review BMC Public Health 2008 8 289 10.1186/1471-2458-8-289 18702821 PMC2533327

[B18] Slama K Chiang C-Y Enarson DA Hassmiller K Fanning A Gupta P Ray C Tobacco and tuberculosis: a qualitative systematic review and meta-analysis The International Journal of Tuberculosis and Lung Disease 2007 11 10 1049 1061 17945060

[B19] Chan-Yeung M Dai DLK Cheung AHK Chan FHW Kam K-M Tam C-M Leung C-C Tuberculin skin test reaction and body mass index in old age home residents in Hong Kong J Am Geriatr Soc 2007 55 1592 1597 10.1111/j.1532-5415.2007.01316.x 17908061

[B20] Forskrift om tuberkulosekontroll (Norwegian)(Regulations on control of tuberculosis) http://www.lovdata.no/cgi-wift/ldles?doc=/sf/sf/sf-20020621-0567.html

[B21] Nasjonalt Folkehelseinstitutt Forebygging og kontroll av tuberkulose, en veileder (Norwegian)(Guidelines for prevention and control of tuberculosis) 2002 7 Oslo: Nasjonalt Folkehelseinstitutt

[B22] Johnsen NL Steen TW Meyer HE Heldal E Skarpaas IJK Bjune G Cohort analysis of asylum seekers in Oslo, Norway, 1987-1995: effectiveness of screening at entry and TB incidence in subsequent years Int J Tuberc Lung Dis 2005 9 1 37 42 15675548

[B23] Harstad I Heldal E Steinshamn SL Garasen H Jacobsen GW Tuberculosis screening and follow-up of asylum seekers in Norway. A cohort study BMC Public Health 2009 9 1 141 10.1186/1471-2458-9-141 19442260 PMC2689201

[B24] Winje BA Oftung F Korsvold GO Mannsåker T Jeppesen AS Harstad I Heier B Heldal E Screening for tuberculosis infection among newly arrived asylum seekers: comparison of QuantiFERON TB Gold with tuberculin skin test BMC Infectious Diseases 2008 8 65 8:65 10.1186/1471-2334-8-65 PMC2405787 18479508

[B25] Harstad I Heldal E Steinshamn SL Garasen H Winje BA Jacobsen GW Screening and treatment of latent tuberculosis in a cohort of asylum seekers in Norway Scand J Public Health 2009 38 3 275 282 10.1177/1403494809353823 19914972

[B26] Sanchez A Gerhardt G Natal S Capone D Espinola A Costa W Pires J Barreto A Biondi E Larouze B Prevalence of pulmonary tuberculosis and comparative evaluation of screening strategies in a Brazilian prison Int J Tuberc Lung Dis 2005 9 6 633 639 15971390

[B27] Cain KP Benoit SR Winston CA Mac Kenzie WR Tuberculosis among foreign-born persons in the United States JAMA 2008 300 4 405 412 10.1001/jama.300.4.405 18647983

[B28] Kempainen R Nelson K Williams DN Hedemark L Mycobacterium tuberculosis disease in Somali immigrants in Minnesota Chest 2001 119 1 176 180 10.1378/chest.119.1.176 11157601

[B29] Wobeser WL Yuan L Naus M Corey P Edelson J Heywood N Holness DL Expanding the epidemiologic profile: risk factors for active tuberculosis in people immigrating to Ontario CMAJ 2000 163 7 823 828 11033709 PMC80504

[B30] Steen TW Farah MG Johnsen NL Johnsen H Johnsen UL [Extrapulmonary tuberculosis among Somali immigrants in Norway](in Norwegian) Tidsskr Nor Laegeforen 2003 123 6 818 821 12693126

[B31] Holmes CB Hausler H Nunn P A review of sex differences in the epidemiology of tuberculosis Int J Tuberc Lung Dis 1998 2 2 96 104 9562118

